# Long-Term Outcomes of Microsurgical Nasal Replantation: Review of the Literature and Illustrated 10-Year Follow-Up of a Pediatric Case with Full Sensory Recovery

**DOI:** 10.3389/fsurg.2015.00006

**Published:** 2015-02-24

**Authors:** Nicholas J. Marsden, Amanda Kyle, Zita M. Jessop, Iain S. Whitaker, Hamish Laing

**Affiliations:** ^1^Welsh Centre for Burns and plastic Surgery, Morriston Hospital, Swansea, UK; ^2^Reconstructive and Regenerative Medicine Research Unit (ReconRegen), Institute of Life Science, College of Medicine, Swansea University, Swansea, UK; ^3^Occupational Therapy Department, Morriston Hospital, Swansea, UK

**Keywords:** nose replantation, artery-only-replantation, composite graft, nasal reconstruction, *Hirudo medicinalis*, sensory recovery

## Abstract

We present a case of successful artery only total nose replantation in an 18-month-old child, with 10 years of follow-up and full sensory recovery despite no nerve repair. The common absence of veins for anastomosis does not prevent successful replant, as demonstrated with the use of *Hirudo medicinalis* use in this unique case. We comprehensively review the literature of this rare and complex injury and advocate microsurgical replantation where possible over other methods of nasal reconstruction.

## Introduction

The nose is the most prominent feature on the human face, critically involved in appearance, both to oneself and to others, and it is involved significantly in the perception of beauty (1). In addition to esthetics, the nose serves physiological functions such as phonation and olfaction. Nasal reconstruction is one of the oldest forms of surgery, dating back to India circa 600 BCE (2). Sushrata described the use of a pedicled forehead flap to reconstruct the nose, which is undoubtedly one of the earliest contributions to reconstructive techniques that are still used in modern day plastic surgery. Total or partial amputation of the nose represents a challenge to even the most experienced of surgeons, with the final result varying greatly depending on the reconstructive techniques used.

In this article, we present a unique long-term follow-up of a case of pediatric artery, only nasal replantation with full sensory recovery and excellent esthetic outcome, along with a review of the literature.

In an age of over proliferation of case reports in the surgical literature, we wanted to focus on the novel aspects of the case. Pediatric nasal replantation is rare but not novel, and there have been several reports in the literature (3–7). Artery only nasal replantation, when no appropriate vein could be identified is a common problem and various methods have been employed to deal with the resulting venous congestion (4, 5, 8–12). We focus on the unusual aspects of the case – the 10-year follow-up, with the age at operation 18 months, the youngest reported in the literature, and the full sensory recovery despite absence of primary neurorrhaphy.

## Background

**Figure 1:**
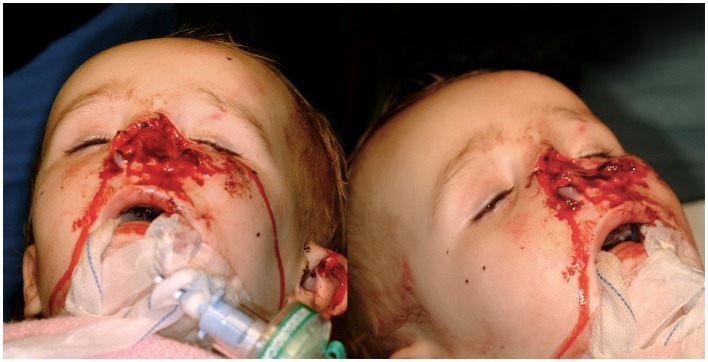
**Pre-operative image of the nasal defect, sparing only a small part of the nasal dorsum, septum, and right alar**.

An 18-month-old boy suffered a traumatic total amputation of the nose following a dog bite. He arrived in the unit with the nasal amputate appropriately transferred and a cold ischemia time of two and a half hours. On examination, the nose was amputated at the level of the piriform aperture with <25% of the nasal dorsum, septum, and section of the right alar remaining (Figure 1). The amputated piece contained the columella, the tip, both soft triangles, the left alar, the left lateral side wall, and around 75% of the right alar, side wall, and dorsum. The patient was immediately taken to theater for consideration of microvascular replantation.

**Figure 2:**
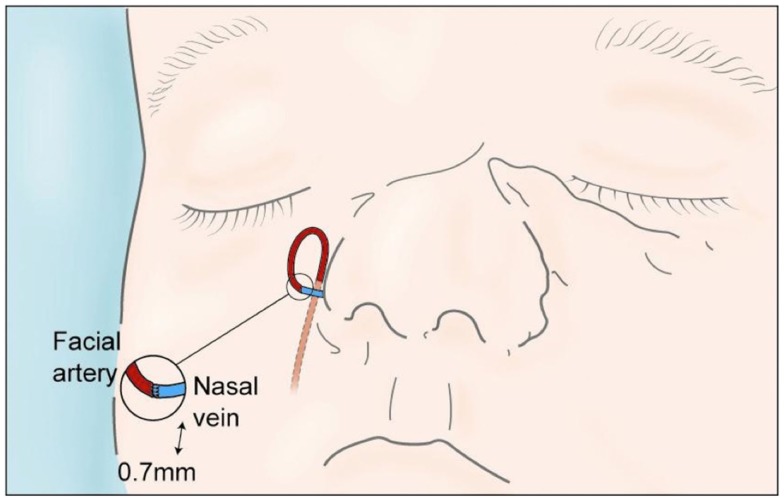
**Artists illustration of the 0.7 mm vein-to-artery microvascular anastomosis**.

**Figure 3:**
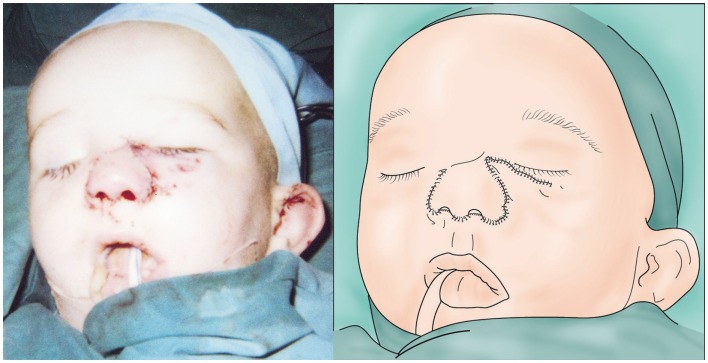
**Immediate post-operative appearance (left) and illustration (right) of the replanted nose showing early evidence of congestion**.

**Figure 4:**
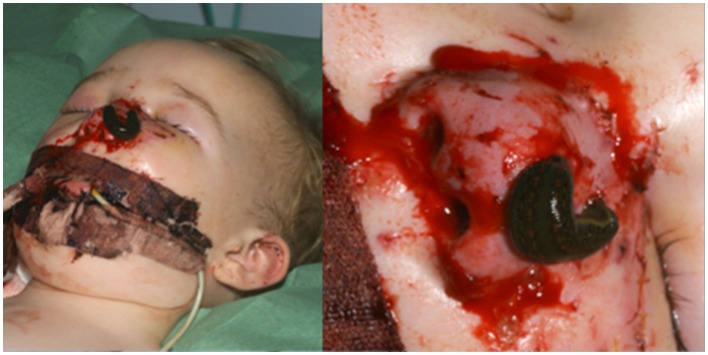
**Day 2 post-replant (left), showing leech application to the congested nose with resulting ooze from bite sites and wound edges (right)**.

On exploration, only one vessel, the right lateral nasal vein, was identified in the amputated part (0.7 mm). Minimal debridement of the wound edges to reduce soft tissue loss and thorough washout with saline was performed. Using an extension of the wound into the right nasolabial fold, the superior facial artery was identified. The artery was divided and reflected caudially and a vein-to-artery microsurgical anastomosis performed using 10/0 Ethilon™(Figure 2). The replanted segment immediately turned pink at the tip, which spread peripherally, with bleeding from the wound edges. The mucosa and cartilage were repaired with 7/0 and 6/0 Vicryl^®^, respectively, and skin was repaired with 6/0 Vicryl rapide™(Figure 3). No nerves were visible and therefore primary neurorrhaphy was not an option. Bilateral nose packs were inserted. Medicinal leeches (*Hirudo medicinalis*) were applied at the end of the procedure as the replanted nose showed signs of venous congestion. The operative time was 4 h and total ischemic time was <6 h. The patient remained intubated and ventilated for 7 days to allow intermittent leech therapy (Figure 4). Leeches were applied as deemed clinically necessary, when the nose showed evidence of venous congestion. Antibiotic prophylaxis with ciprofloxacin was continued for the duration of leech therapy based on departmental guidelines and the patient required a total of 1000 ml of packed red blood cells transfused over a 4-day period post-injury. He was discharged on day 14 and was closely followed up in clinic, with regular clinical photographs.

**Figure 5:**
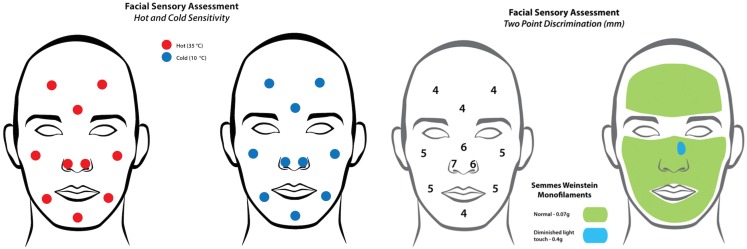
**Results of the temperature sensitivity (left) and two-point discrimination tests (right) demonstrating full sensory recovery despite absence of primary neurorrhaphy**.

**Figure 6:**
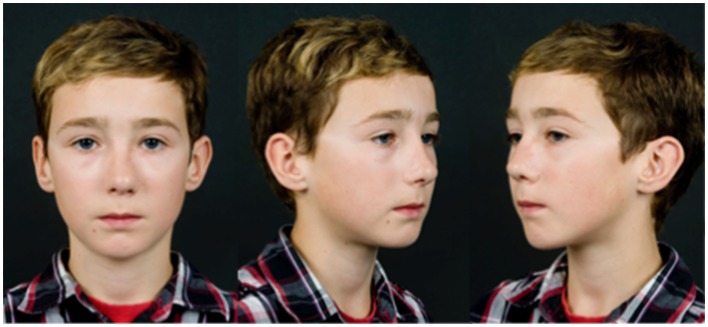
**Ten-year follow-up showing excellent cosmetic result**.

Full facial sensory assessment, including threshold pressure, two-point discrimination (TPD) and hot and cold identification was performed 10 years following the injury by an advanced practitioner in occupational therapy (Figure 5). Threshold pressure sensation, tested with the Semmes Weinstein Monofilaments (SWM), had a normal positive response of 0.07 g to all areas except a small 1.5 cm × 1 cm area to the left side of the replanted skin, which was positive to 0.4 g of pressure. Static TPD, assessed using a discriminator, revealed a reduced static TPD of the replanted skin (6 mm left and 7 mm right) compared to unaffected areas (5 mm chin and 4 mm forehead), although these fall within normative data of 8 mm published for TPD of the nose (13). Temperature sensation tested using thermometer regulated 2 cm diameter blunt metal rods, was 100% accurate, with identification of warm (30°C) and cold (10°C) stimulus to within 2 cm. On questioning, the patient reported no symptoms of hypersensitivity or cold intolerance. He had an excellent cosmetic result, without the need for any revision surgery and no nasal airflow obstruction (Figure 6). Nasal growth was unaffected and facial proportions were normal in terms of horizontal facial thirds and nasal wing base equaling the intercanthal distance, and he had appropriate projection of the nose and nasal tip according to Crumley and Lancers 5:4:3 ratio (14).

## Discussion

Reconstruction following nasal amputation has been attempted for over 2000 years. The autologous treatment options of these defects have advanced from replacement as a composite graft to microvascular replantation.

### Composite grafts

The largest reported series dates back to 1836 by Hoffacker who was an attending physician to the Heidelberg dueling matches. He reported 12 successful replants as composite grafts out of 16 patients (15). The success rate of replacement of nasal parts as composite grafts is varied. The maximum diameter of the graft should not exceed 1.25 cm × 1.5 cm (5, 10) as the success depends on the amount of raw surface available for revascularization, which is limited due to the tri-dimensional shape of the nose. The mechanism of injury and the condition of the graft are also important, with the success reduced with macerated and crushed tissues, and the timing should ideally be within 2 h of injury (15). Several methods have been proposed to improve survival of composite auricular grafts in nasal reconstruction, the most common being corticosteroid use, cooling, and hyperbaric oxygen therapy. One review suggests the use of a reducing dose of corticosteroids and post-operative cooling for 48–72 h to improve graft survival (16); however, there is no literature regarding the success of these methods in composite grafting of nasal tissues following traumatic injury. Further high-level evidence research is needed in this area before clear recommendations can be produced. The senior author suggests that in the absence of suitable vessels for replantation, composite grafting should be attempted in pediatric patients, irrespective of the size of the amputated part, as even partial survival will reduce the defect for autologous reconstruction at a later date.

### Replantation

The advances in microsurgical techniques have allowed facial tissues to be replanted successfully (17). Since James first performed successful microvascular nasal replantation in 1976 (3), there have been 19 further cases reported in the literature (Table 1). Seventeen-reported total survival, with three requiring further reconstructive surgeries for partial necrosis (3, 7, 19). The most difficult challenge in nasal replantation is identifying an appropriate donor vein for anastomosis. In artery only procedures, there are numerous methods described to reduce venous congestion. The uses of medicinal leeches (4, 5, 8, 9, 11, 12, 23) when available are an excellent method of venous decompression, as demonstrated in our case. It is important to note that antibiotic prophylaxis (most commonly ciprofloxacin) should be employed during leech therapy, due to the risk of *Aeromonas hydrophila* infection. Other methods include pinpricking or tip abrasion with heparinized sponge wiping (6, 7, 10, 11, 18, 20, 22, 25), intravenous heparin and dextran (3, 7, 8, 11, 18, 20, 22), oral aspirin (3, 8, 9, 11, 12, 18, 22), and intra-replant injections of heparin (12, 18, 19), or a combination of the above. Due to the very young age of the patient in the case discussed, elective intubation was continued for the duration of leech therapy. Prolonged intubation carries known risks such as ventilator acquired pneumonia, volume overload, and pneumothorax; however, this risk is low in periods <21 days. It was decided by a multidisciplinary team that the risk of the patient not tolerating regular leech therapy while awake, and resultant loss of the replant, outweighed the small risk associated with a short period of mechanical ventilation in this case.

**Table 1:** **Summary of the previous 20 cases of nose replantation reported in the literature, highlighting the injury details, microsurgical techniques used, and outcomes**.

Reference	Patient age/sex	Injury size	Anastomosis (A = arterial V = venous)	Post-operative management	Results (follow-up duration)	Sensory recovery
James (3)	3 year-old female	5 cm × 3 cm	A–A V–V	IV heparin/dextran, oral aspirin.	Partial necrosis (7 months)	N/A
Tajima (7)	9 year-old female	Sub-total amputation – 2/3 lower nose	A–A (+ vein graft) A–V (+ vein graft)	IV heparin/dextran. Pin-pricking and heparin-soaked gauze	Partial necrosis – 3 revision surgeries (20 months)	N/A
Niazi (5)	10 year-old male	Sub-total amputation – 2/3 nose	A–A No V	Leech	Total survival – hypertrophic scar (9 months)	N/A
Jeng (18)	30 year-old male	Sub-total amputation 3.5 cm × 3.5 cm	A–A A–V (+ vein graft)	IV heparin/dextran, oral aspirin. Pin-pricking	Total survival – excellent cosmesis (1 year)	N/A
Sanchez-Olaso (10)	18 year-old male	Amputation of nasal tip including full thickness of alar cartilages	A–A No V	Open venous drainage (5 days) then pin pricking	Total survival (15 months)	Protective sensation
Jeng (19)	55 year-old male	3 cm × 3 cm	A–A No V	Intra-replant heparin	Partial necrosis – required revision surgery (2 years)	N/A
Hussain (20)	28 year-old male	Sub-total amputation of right alar (2.5 cm × 1.5 cm)	A–A V–V	IV heparin/dextran. Intra-replant heparin injection and pin-pricking	Total survival (6 months)	N/A
Yao (21)	49 year-old male	Total amputation (4 cm × 4 cm)	A–A V–V	None described	Total survival (3 months)	8 mm TPD
Hammond (21)	15 year-old male	Total amputation	A–A × 2 V–V	Tip abrasion + heparinized sponge wiping	Total survival – excellent cosmesis (6.5 years)	Normal with SWM testing
Kayikcioglu (11)	46 year-old female	Near-total amputation (4 cm × 5 cm)	A–A No V	IV heparin/dextran, oral aspirin/dipyridamole. Pin-pricking, leeches	Total survival – good cosmesis (6 months)	N/A
Akyurek (22)	40 year-old	Left alar amputation with 2 mm skin bridge (2.5 cm × 1 cm)	A–A No V	IV heparin/dextran, oral aspirin. Pin-pricking and heparinized sponge wiping	Total survival – excellent cosmesis (3 months)	8 mm TPD
Flores (8)	54 year-old male	Heminasal amputation	A–A No V	IV heparin, oral aspirin, and leeches	Total survival – excellent cosmesis (1 year)	N/A
Kim (23)	48 year-old male	Partial amputation (2.5 cm × 2.6 cm)	A–A V–V	Leeches	Total survival (77 days)	10 mm TPD
Okumus (24)	34 year-old male	Nasal tip amputation	A–A V–V	None described	Total survival (not documented)	N/A
Sun (25)	38 year-old female	Sub-total amputation (3 × 3)	A–A × 2 A–V	Pin-pricking	Total survival – cicatricial healing to tip (not documented)	N/A
	39 year-old female	Total amputation (3 × 4)	A–A × 2 V–V	None described	Total survival (not documented)	N/A
Stillaert (4)	66 year-old female	Sub-total amputation (3.5 × 2.5)	A–A No V	Leeches	Total survival (7 months)	N/A
	10 year-old male	Sub-total amputation, connected by fibrofatty bridge	A–A No V	Leeches	Total survival (5 months)	N/A
Anderson (12)	41 year-old female	Sub-total amputation (4 × 4.5)	A–A No V	Intra-replant tinzaparin, oral aspirin, leeches	Total survival (6 month)	N/A
Gilleard (9)	36 year-old male	Sub-total amputation (3.5 × 4)	A–A (+ vein graft) V–V (+ vein graft)	Aspirin, tinzaparin, and leeches	Total survival (3/12)	N/A

Nearly 50% of all reported cases were artery only, with no venous anastomosis (3, 5, 8, 10–12, 20, 22), and of these eight of the nine patients had total survival. In the 11 cases where both arterial and venous anastomosis were completed, three required interpositonal vein grafts, which increased the complexity of the case and reported operating times (7, 9, 18). In the case presented, only a single vein was identified in the amputated part, which was anastomosed to a branch of the facial artery. This is the first report of an arterialized venous nasal replant in the literature, although success has been reported in digital replantation after single arterio-venous anastomosis (26). The exact mechanism of survival in these replants is not fully understood, although theories of arterio-venous shunting and reverse flow of blood from the venules into the capillaries have been suggested (27). Venous congestion is inevitable and requires the use of the previously described methods to relieve congestion, and failure rates are as high as 20–50% have been reported (27).

Bite injuries not only pose an increased infection risk but also due to the crush-avulsion type mechanism, are notorious for increased tissue damage compared to guillotine type injuries. Despite the increased tissue trauma inflicted from bite injuries, there are several reports of successful nasal replantation following animal and human bites (3, 4, 8, 9, 12). As in our case, with thorough washout, minimal debridement, and antibiotic coverage, excellent cosmetic results can be achieved despite this challenging mechanism of injury and bite wounds should not be a contraindication to replantation.

The child in our case required a total of 1000 ml transfusion over the post-operative period. Transfusion requirements have been noted in lip, ear, and scalp replantations (28, 29) and were reported in four of the previous cases of nasal replantation (6, 9, 11, 12). Every attempt at achieving venous anastomosis should be made to reduce the need for alternate venous drainage techniques, which can lead to significant blood loss. The resultant risk of disease transmission from the blood product transfusion must be weighed up against other methods of reconstruction that do not run the risk of transfusion requirements. We feel that the functional and cosmetic outcomes of nasal replantation are of huge benefit in comparison to the risk of disease transmission after transfusion, although every effort should be made to attempt venous anastomosis.

### Sensory recovery

Sensory recovery after nasal replantation is rarely reported satisfactorily in the literature. Only five previous cases describe any form of sensory recovery at follow-up, of which one reports subjectively that protective sensation was regained (10) without formal assessment. The most common assessment reported was TPD. Yao (21) and Akyurek (22) both reported outcomes of 8 mm for TPD, which falls within the normal range for the nose; however, they give no indication of how this compared to other facial areas. Hammond (6) used SWM testing to report full sensory recovery; however, they did not give any further details with regards to specific results of threshold pressure measurements. In our case, we report a full assessment including, threshold pressure, static TPD, and temperature, with full sensory recovery. No nerve repair was performed in our case, or in any of the previous cases in the literature. In view of this, attempting a primary neurorrhaphy at the time of replantation is not necessary to achieve a satisfactory sensory outcome.

## Concluding Remarks

Our case demonstrates that successful total nasal replantation in very young children can be successful in terms of sensory and cosmetic outcomes despite unfavorable conditions. Bite injuries, extremes of age, and absence of veins for anastomosis should not be considered as contraindications to performing microvascular replantation, as success will give far superior results to delayed multi-stage reconstruction.

## Author Contributions

All authors made substantial contributions to the conception, design, and completion of the work and have approved the final manuscript.

## Conflict of Interest Statement

The authors declare that the research was conducted in the absence of any commercial or financial relationships that could be construed as a potential conflict of interest.
